# Editorial: Nutritional management for the development and gut health of young ruminants

**DOI:** 10.3389/fvets.2022.1042848

**Published:** 2022-09-30

**Authors:** Zhixiong He, Kefyalew Gebeyew, Morteza Hossieni Ghaffari

**Affiliations:** ^1^CAS Key Laboratory for Agro-Ecological Processes in Subtropical Region, National Engineering Laboratory for Pollution Control and Waste Utilization in Livestock and Poultry Production, Hunan Provincial Key Laboratory of Animal Nutritional Physiology and Metabolic Process, Institute of Subtropical Agriculture, The Chinese Academy of Sciences, Changsha, China; ^2^Hunan Co-innovation Center of Animal Production Safety (CICAPS), Changsha, China; ^3^University of Chinese Academy of Sciences, Beijing, China; ^4^Institute of Animal Science, University of Bonn, Bonn, Germany

**Keywords:** gut health, early nutrition, microbial community, ruminants, weaning stress

Early weaning is a common practice in modern animal husbandry to promote adult reproductive performance and reduce costs associated with feeding milk replacers. It is the most challenging phase of life for young animals, as evidenced by a high incidence of diarrhea, distress, intestinal disorders, mortality, growth failure, and other health disorders (1). The process of weaning, along with the immature gut and immune system, makes young animals more susceptible to disease. The feeding regime and diet during this period have a persistent, long-term effect on gut maturation and thus on future production performance and health (2). In young ruminants, energy derived from glucose in a milk-based diet is absorbed through the columnar epithelium in the intestine prior to weaning. After weaning, however, most of the energy is absorbed in the form of short-chain fatty acids (SCFA) *via* the stratified squamous epithelium of the rumen, where the solid diet can be converted to SCFA by microbial fermentation in the rumen. Therefore, an appropriate nutritional strategy to promote structural and functional development of the gut is critical for survival and growth of young ruminants later in life.

The aim of this Research Topic was to compile original papers that could contribute to the current knowledge and understanding of gut structural and functional development and nutritional interventions to improve survival and growth of young ruminants at critical stages. For this Research Topic, seven original papers were compiled.

Nutritional intervention prior to weaning is critical to ensure that neonates can maintain physiological development when exposed to the stress associated with weaning. In addition to nutritional intervention, some management approaches, such as providing adequate space and peers for social interactions, can play a critical role in promoting wellbeing and microbial development during this period (3). Palma-Hidalgo et al. investigated the effects of rearing goat kids with and without goat peers on gut functional development and microbial composition. The results showed that goat kids raised with goats of the same age had a greater diversity of bacterial communities and a transfer of protozoa and specific methanogens, demonstrating the role of early social contact in promoting gut microbial development. On the other hand, weaning stress could disrupt the digestive and absorptive capacity of the neonatal gut by inducing tremendous changes in gut structure. Therefore, understanding the mechanism of intestinal damage induced by various weaning strategies is critical for effective nutritional intervention. In this regard, using goats as experimental animals, Han et al. investigated the molecular regulatory mechanism of intestinal damage in neonates fed either milk replacer or sheep milk after receiving colostrum. The authors found that the peroxisome proliferator-activated receptors (PPARs) pathway and ferroptosis may be involved in weaning stress-induced intestinal injury or structural changes in newborn lambs using multi-omics approaches. This opens another view on the role of these two signaling pathways in intestinal damage caused by weaning stress.

Feeding functional feed supplements to newborn ruminants is important to alleviate stress associated with weaning and to promote metabolic and physiological development or maturation (4). Tian et al. showed that the addition of polysaccharides from wheat bran to milk replacer improved overall growth and rumen development, which was associated with a change in the structure of the rumen bacterial community. Similar results were observed by Wang et al. who showed that the addition of anthocyanins from purple corn to a staple diet during the heat stress period altered the structure of rumen bacteria and increased antioxidant capacity and volatile fatty acid production in goats. The development of novel feeds highlights the need for further research to gain clear insight into the mode of action responsible for the phenotypic responses.

Feeding a high concentrate level is a common practice to achieve rapid growth and high production efficiency on modern livestock farms. This feeding practice adversely affects the gut microbiota and luminal environment (5), which play a critical role in gut health and maturation. The study by Wang et al. showed that feeding a rice diet containing 90% concentrate altered the ratio of bacterial communities toward unpleasant patterns. The observed lower pH, higher plasma free lipopolysaccharides, and volatile fatty acid (VFA) concentrations could be related to the altered composition of the microbiota in response to feeding diets high in paddy. Although the increased VFA improves energy supply from the appendix, the lower pH affects the epithelium of the appendix because of its limited buffering capacity compared with the rumen.

In addition to nutrition, the feeding regime may also affect the gut microbiota, which may interact with the host. These effects could be due to differences in the nutrient composition of feeds under different feeding paradigms. In this Research Topic, Shah et al. examined the diversity of the fecal microbiota of yaks raised on natural pastures and on fattening farms. The results showed that grazing on natural pastures promoted the accumulation of fecal bacteria associated with fiber degradation. In contrast, bacteria associated with protein and carbohydrate degradation dominated the feces of goats raised on the feeding system, underscoring the influence of the feeding paradigm on microbiota community structure and function. Nevertheless, the study by Zhang et al. investigated seasonal differences in the fecal microbiota of Chinese Merino fine wool sheep. The authors indicated that the composition of the microbiota changes across the four seasons, with greater diversity being observed in summer. These changes in microbiota structure in different seasons could allow physiological adaptation of goats.

In conclusion, promising results were observed in the functional and microbial development of the gut in response to different nutritional management during the pre- and post-weaning periods.

## Author contributions

All authors listed have made a substantial, direct, and intellectual contribution to the work and approved it for publication.

## Funding

This work was jointly supported by Chinese Academy of Sciences (Strategic Priority Research Program Grant NO. XDA26050102 and XDA26040304), the National Natural Science Foundation of China (32072760), and the Natural Science Foundation of Hunan Province of China (2022JJ10054).

## Conflict of interest

The authors declare that the research was conducted in the absence of any commercial or financial relationships that could be construed as a potential conflict of interest.

## Publisher's note

All claims expressed in this article are solely those of the authors and do not necessarily represent those of their affiliated organizations, or those of the publisher, the editors and the reviewers. Any product that may be evaluated in this article, or claim that may be made by its manufacturer, is not guaranteed or endorsed by the publisher.
